# Difficulties in the management of the meningitis with *C. neoformans*

**DOI:** 10.1186/1471-2334-14-S4-P36

**Published:** 2014-05-29

**Authors:** Elisabeta Otilia Benea, Doina Niculescu-Lupu, Șerban Benea, Daniela Camburu, Manuela Podani, Mihaela Ionica, Cozmina Andrei

**Affiliations:** 1 National Institute for Infectious Diseases “Prof. Dr. Matei Balş”, Bucharest, Romania; 2 Carol Davila University of Medicine and Pharmacy, Bucharest, Romania

## 

The management of the meningitis with *C. neoformans* raises many problems: the choosing of the appropriate antifungal therapy, the prevention and/or the control of the complications, the correct management of the antiretroviral therapy.

We report two cases with HIV infection same immunological and clinical stage, the first of them with relatively recent infection, the second with long-term infection (>20 years) and antiretroviral therapy - experienced, but discontinued for 4 years, who developed the same opportunistic infection: cryptococcal meningitis.

Although immunological status was similar in both patients, cerebrospinal fluid inflammatory response was stronger in patient infected latest; both had increased cerebrospinal fluid pressure, requiring repeated lumbar punctures.

The antifungal treatment algorithm was applied according to guidelines, to availability of medications at that time and antifungal susceptibility testing results.

Although the combination of fluconazole plus flucytosine is knowed as being clinically inferior to amphotericin B–based therapy, faster rate of cerebrospinal fluid sterilization was seen in patient with greater cerebrospinal fluid inflammatory response rather than in patient receiving antifungal therapy considered as “gold standard”.

To avoiding the immune reconstitution syndrome, antiretroviral therapy was initiated in both cases after more than 4 weeks of therapy of opportunistic infection (after 2 weeks of sterilizing cerebrospinal fluid cultures); however, the patient with long-term HIV infection developed immune reconstitution syndrome after 21 days of initiating therapy.

Choosing antiretroviral therapy was achieved in both cases according to guidelines, depending on the patient's medical history (including previous regimens therapy) and drug interactions.

Our cases illustrate that the same disease can have different solutions because the patient makes the difference.

